# The Role of Hyperglycemia in Endometrial Cancer Pathogenesis

**DOI:** 10.3390/cancers12051191

**Published:** 2020-05-08

**Authors:** Frances L. Byrne, Amy R. Martin, Melidya Kosasih, Beth T. Caruana, Rhonda Farrell

**Affiliations:** 1 School of Biotechnology & Biomolecular Sciences, Faculty of Science, University of New South Wales, Sydney 2052, Australia; beth.caruana@gmail.com; 2 School of Women’s and Children’s Health, Faculty of Medicine, University of New South Wales, Sydney 2052, Australia; amyrmartin8@gmail.com (A.R.M.); m.kosasih@student.unsw.edu.au (M.K.); 3 Prince of Wales Private Hospital, Randwick, NSW 2034, Australia; rhondafarrell@mac.com; 4 Chris O’Brien Lifehouse, Camperdown, Sydney 2050, Australia

**Keywords:** hyperglycemia, HbA1c, glucose metabolism, uterine cancer, metformin

## Abstract

Endometrial cancer is one of the most common cancers in women worldwide and its incidence is increasing. Epidemiological evidence shows a strong association between endometrial cancer and obesity, and multiple mechanisms linking obesity and cancer progression have been described. However, it remains unclear which factors are the main drivers of endometrial cancer development. Hyperglycemia and type 2 diabetes mellitus are common co-morbidities of obesity, and there is evidence that hyperglycemia is a risk factor for endometrial cancer independent of obesity. This review aims to explore the association between hyperglycemia and endometrial cancer, and discuss the evidence supporting a role for increased glucose metabolism in endometrial cancer and how this phenotype may contribute to endometrial cancer growth and progression. Finally, the potential role of blood glucose lowering strategies, including drugs and bariatric surgery, for the treatment of this malignancy will be discussed.

## 1. Endometrial Cancer

Endometrial cancer (EC) is an adenocarcinoma that originates from the epithelial cells lining the uterine cavity. The tumor microenvironment surrounding these cells comprises stromal cells, endothelial cells [1] and many different types of immune cells [2], all of which can influence cancer progression and response to treatment. Although most ECs are early stage and confined to the uterus, others spread by invading the myometrium and metastasizing to distant sites such as lymph nodes, liver and lung [3]. According to GLOBOCAN 2018 statistics, EC is the sixth most common cancer and the 11th leading cause of cancer death in women worldwide, with 382,069 new cases and 89,929 deaths [4].

### 1.1. Classification of Endometrial Cancers

EC has been classically categorized into two clinicopathological subtypes; Type I and Type II. Type II tumors are generally more invasive, estrogen and progesterone receptor (ER/PR) negative and confer a poor prognosis; but account for less than 15% of all cases [5,6]. Type II ECs include grade 3 endometrioid, papillary serous, clear cell, and carcinosarcoma histologies [7]. In contrast, most EC cases are Type I tumors that are frequently low grade endometrioid tumors, confined to the uterus, ER/PR positive, and have higher survival rates following treatment with primary surgery [6]. Since most EC cases are Type I, the majority of studies sited in this review refer to or contain data from Type I EC, unless otherwise stated. However, recent genetic investigations have supported re-classification of EC into 4 molecular subgroups; (1) DNA-polymerase epsilon (POLE) (ultramutated), (2) microsatellite instability hypermutated, (3) copy-number low (microsatellite stable), and (4) copy-number high (serous-like) [8]. Each subgroup has prognostic significance, with the POLE group having the best prognosis (>95% progression-free survival) and the copy-number high group the worst (5-year progression-free survival of 50%). These molecular subgroups are associated with body mass index (BMI), with women in the POLE cluster having the lowest BMI and those in copy-number low cluster having the highest BMI, suggesting that obesity may impact the genetic landscape of endometrial tumors [9].

### 1.2. Risk Factors for Endometrial Cancer

Recent epidemiological studies have shown an increasing incidence of EC, especially in countries with rapid socioeconomic transitions [10]. The rate of new cases of EC is expected to rise due to an aging population and an increased prevalence of risk factors, particularly obesity [11]. In addition to obesity, a number of factors have been attributed to an increased likelihood of developing EC, including but not limited to; advancing age, late onset menopause, lower age of menarche, chronic anovulation including polycystic ovarian syndrome, estrogen therapy in the absence of progesterone, tamoxifen therapy, hereditary predisposition (Lynch syndrome), and nulliparity [12,13,14,15]. Several of these factors influence the length of time and level of exposure the uterus has to estrogen and progesterone.

A number of Mendelian Randomization (MR) studies have identified causal factors for EC (as reviewed [16,17]). MR is an analytical method that uses genetic determinants (variants), typically single nucleotide polymorphisms (SNPs), as instrumental variables for a modifiable risk factor. The MR approach is considered to be less affected by confounding factors or reverse causation but is dependent on assumptions [17]. One study showed that a genetically-predicted increase in age of menarche, adjusted for genetically-predicted body-mass index (BMI), was associated with a lower risk of EC [18]. Another showed that variants in *CYP19A1* (gene that encodes aromatase/estrogen synthetase) were associated with increasing estradiol levels in post-menopausal women, and risk of EC, in women of European ancestry [19]. SNPs associated with obesity (BMI), but not waist:hip ratio, were also shown to be associated with EC, indicating that obesity is a causal factor for EC [20]. Genetically-predicted higher fasting insulin levels (using 18 SNP variants) and post-challenge insulin levels (using 17 SNP variants), but not fasting glucose (using 36 SNP variants) or Type 2 diabetes (using 49 SNP variants), were associated with increased risk of EC [21] (Table 1). A more recent MR study by O’Mara et al. included the most numbers of cases and controls to date; 12,906 endometrial cancer cases and 108,979 country-matched controls of European ancestry [22]. This study confirmed previous findings (higher BMI associated with increased EC risk and later menarche with lower EC risk) and demonstrated that the protective effect of later menarche is partially mediated by the known relationship between lower BMI and this factor [22]. Overall, these genome-wide association studies may provide vital information to those proposing the development of a risk prediction scoring system for women at high risk of EC [23]. A scoring system such as this could enable prophylactic treatment to reduce the incidence of EC, particularly those with Type I EC [23].

#### 1.2.1. Links between Obesity and Endometrial Cancer

Worldwide, the prevalence of obesity [body mass index (BMI) > 30 kg/m^2^] in women has increased fivefold in the last four decades [40]. In women, it is estimated that 20% of all cancer-related deaths are due to obesity, and of these, EC is the most strongly associated [41,42]. EC has the strongest association with obesity of all malignancies with a population attributable fraction (PAF) of 42.4% in the Oceania population (including Australia and New Zealand) and 56.8% in the US population [43,44]. Obese women are 2–3 times more likely to be diagnosed with EC [45] and the age of diagnosis of EC is inversely correlated with BMI [46]. Each 5 kg/m^2^ increase in BMI correlates to a large increase in EC risk, with most observational studies reporting a 200%–400% increased risk of developing EC in individuals with BMI ≥25 kg/m^2^ [47]. Calle et al. also reported a 6.25-fold increased risk of uterine cancer-related death for morbidly obese women compared to those within normal range of BMI [41]. Bariatric surgery is an effective treatment for weight loss for morbidly obese patients and a scoping review by Aubrey et al. found EC risk reduction in obese women who underwent bariatric surgery [48]. Bariatric surgery as an intervention to reduce EC risk will be further discussed later in this review (see Section 3.2).

Mechanisms linking obesity and cancer have been described in the literature [49,50]. Several of these have been proposed to link obesity to EC development and progression, including: (1) excess estrogen through aromatization of androstenedione to estradiol by adipose-derived aromatase [51], (2) altered secretion of adipokines by adipocytes, specifically lower levels of adiponectin and higher levels of leptin [52] and visfatin [53,54], (3) insulin resistance with associated hyperinsulinemia, increased insulin-like growth factor 1 (IGF-1) and decreased IGF binding protein 1 (IGFBP-1) and sex hormone binding globulin (SHBG) [44], and (4) chronic low grade inflammation from increased levels of proinflammatory cytokines [55,56]. These mechanisms linking obesity and endometrial carcinogenesis are described elsewhere [57,58]. The role of obesity, as a component of metabolic syndrome in EC, is also described in-depth in another review [59].

#### 1.2.2. Links between Hyperglycemia and Endometrial Cancer

Disorders associated with hyperglycemia (Type I and II diabetes mellitus) have an increased risk of EC, indicating that poor control of blood glucose may be an important contributor to the growth of these tumors in women. Three separate meta-analyses on this topic have demonstrated that diabetes mellitus is significantly associated with a twofold risk of developing EC [60,61,62] and several epidemiology studies have also demonstrated that this association is independent of obesity [60,63,64,65] (Table 1). A case-control study involving 942 cases and 1721 controls conducted by Zhang et al. demonstrated a twofold increase in EC risk in women with type II diabetes mellitus (T2DM) compared to their non-diabetic counterparts [66]. Furthermore, hyperglycemia has been associated with EC independent of obesity [67]. In a previous study by Modesitt et al. comparing women with comparable morbid obesity levels with and without Type I EC, circulating glucose levels were higher in women with cancer (119.5 vs. 90.7 mg/dl for non-cancer; *p* = 0.049) [32] (Table 1). Interestingly, other serological factors, including estrogen and insulin, were not significantly different between the two groups [32]. Several large prospective cohort and case-control studies have also found an increased risk of EC with higher blood glucose levels [24,25,27,28,29,30,31,34,35] (summarized in Table 1), although the strength of these associations varies according to BMI, age, and menopausal status in some populations [24,25,27] (Table 1). One observational study did not find an association, however diabetic patients and patients with a fasting blood glucose ≥6.9 mmol/L at baseline were excluded [26] (Table 1). Observational studies are susceptible to confounding and as such, it is possible that hyperinsulinemia, rather than hyperglycemia, is responsible for the association between T2DM and EC risk, as supported by an MR study [21] (Table 1).

Glycosylated hemoglobin (HbA1c) is used as an indicator of blood glucose levels over the preceding 3 months [68]. In Australia, levels ≥6.5% are considered elevated [69]. While HbA1c is a more helpful indicator of long-term glycemic control than fasting or random blood glucose levels, few studies have examined the association between elevated HbA1c and EC (Table 1). However, two small case-control studies showed higher mean HbA1c in EC cases versus controls [36,39]; and a prospective cohort study in a predominantly Maori population (the Indigenous people of New Zealand) found a four-to-five-fold increase in EC risk with elevated HbA1c [38](Table 1). Overall, there is evidence to suggest that the chronic elevation of blood glucose may increase the risk of EC.

## 2. Glucose Metabolism in Endometrial Cancer

### 2.1. Genetic Landscape of Endometrial Cancer Promotes Glucose Metabolism

**Figure 1:**
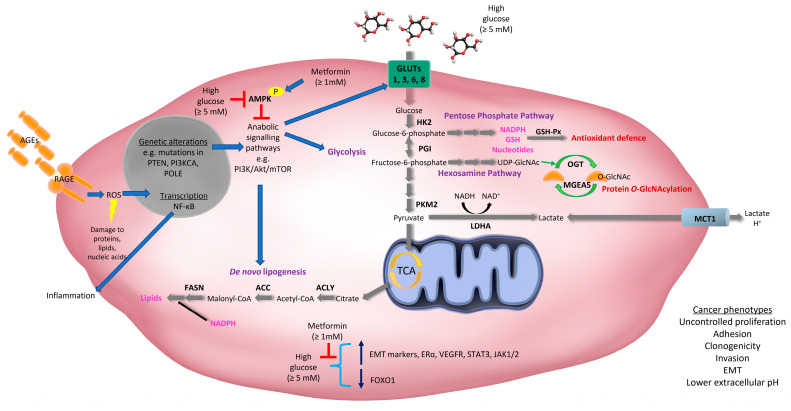
Increased glucose metabolism facilitates endometrial cancer phenotypes. Genetic abnormalities, such as loss of the tumor suppressor, phosphatase and tensin homologue (PTEN), and mutations in phosphatidylinositol 3-kinase (PI3K) family members, are common in EC. Chronic exposure to high blood glucose levels (hyperglycemia) facilitate tumor growth by providing a carbon source for diverse metabolic pathways. Genetic abnormalities common in EC converge on signaling pathways that fuel anabolic growth, particularly the PI3K/Akt/mTOR pathway. This pathway regulates the activity and expression of proteins that control glucose uptake, glycolysis, and de novo lipogenesis. Glucose transporters (GLUTs 1, 3, 6 and 8), enzymes that regulate glycolysis [hexokinase 2 (HK2), pyruvate kinase M2 (PKM2), and lactate dehydrogenase A (LDHA)] and de novo lipogenesis [ATP citrate lyase (ACLY), acetyl CoA carboxylases (ACCs), fatty acid synthase (FASN)] are all elevated in EC. Glucose can also be metabolized via the Pentose Phosphate Pathway (PPP) which facilitates nucleotide biosynthesis, as well as antioxidant defense through the generation of reduced nicotinamide adenine dinucleotide phosphate (NADPH) which is an electron donor for the production of reduced glutathione (GSH). GSH is utilized by glutathione peroxidase (GSH-Px), which is upregulated in EC and promotes antioxidant defense. Monocarboxylate transporter 1 (MCT1) is upregulated in EC and is a proton-coupled symporter that transports lactate inside and outside the cell. Protons transported outside the cell can contribute to a lower extracellular pH. Glucose can also be metabolized through the hexosamine pathway which generates uridine diphosphate N-acetylglucosamine (UDP-GlcNAc). UDP-GlcNAc can be used for the common post-translational modification, O-Linked β-N-acetylglucosamine (O-GlcNAc). The expression of O-GlcNAc transferase (OGT) and Meningioma-Expressed Antigen 5 (MGEA5) are increased in EC, suggesting that protein O-GlcNAcylation reactions may also contribute to EC. Glucose can also bind non-enzymatically with amino groups on proteins to form precursors of advanced glycation end-products (AGEs). Hyperglycemia results in the chronic accumulation of AGEs which when bound to the Receptor for AGE (RAGE) increase inflammatory signaling (by promoting NF-κB activation) and reactive oxygen species (ROS) that can damage cellular proteins, DNA, and lipids. RAGE expression is elevated in EC. EC cells cultured in high glucose conditions have lower phosphorylation of 5’ adenosine monophosphate-activated protein kinase (AMPK), increased expression of epithelial-mesenchymal transition (EMT) markers, estrogen receptor ɑ (ERɑ), vascular endothelial growth factor receptor (VEGFR), signal transducer and activator of transcription 3 (STAT3) and Janus kinases JAK1/2, and decreased expression of forkhead box class O1 (FOXO1). Many of these markers are reversed when EC cells are exposed to supraphysiological concentrations of the anti-diabetic agent, metformin. Mutations in the exonuclease domain of the DNA polymerase POLE confer a favorable prognosis in EC and are linked to higher expression of glycolytic enzymes, including phosphoglucose isomerase (PGI). Glucose can also be metabolized by cells in the tumor microenvironment, including tumor-associated macrophages (TAMs), stromal cells, and bacteria. These cells can also utilize or are affected by lactate produced by EC cells. These other cell types may promote EC initiation and/or progression and resistance to therapy.

Many of the somatic drivers of EC development in some way or another regulate cell metabolism [6,8,70]. These include mutations, amplifications, and over-expression of key regulators of glucose metabolism, particularly phosphatase and tensin homologue (PTEN) and phosphatidylinositol 3-kinase (PI3K)-Protein kinase B (Akt) family members, which are more common in Type I/endometrioid EC (as reviewed [6]) (Figure 1). Traditionally, Type I EC was characterized by the loss of the tumor suppressor, PTEN [71]; a genetic feature also observed in precancerous lesions [72]. PTEN is a lipid phosphatase that inhibits the PI3K-Akt pathway and loss of PTEN leads to activation of Akt, and other downstream targets, such as Rac1 and CDC42, that play important roles in cell cycle progression, migration and invasion [73]. Cell autonomous activation of the PI3K/PTEN/Akt pathway in uterine epithelial cells, via loss of PTEN or activation of Akt, is sufficient to initiate EC in mice [74]. These results suggest that loss of PTEN is a potential initiating factor for EC. Recent studies have shown the PI3K/Akt pathway is altered in up to 93% of Type I ECs, which is likely due to loss of PTEN and mutations in PI3K family members [6,8] (Figure 1).

Other aberrations frequently observed in EC include mutations in V-Ki-ras2 Kirsten rat sarcoma viral oncogene homolog (KRAS) (Type I EC), over-expression of epidermal growth factor receptor (EGFR) (Type I and II EC), nuclear localization of β-catenin (mostly Type I EC), loss of liver kinase B1 (LKB1) and tuberous sclerosis 2 (TSC2) (Type I EC), and mutations in TP53 (mostly Type II EC) [6,75,76]. Alterations in the expression or activity of these proteins stimulate glucose metabolism by various mechanisms, including through the regulation of glucose transporters (GLUTs) and altering the activity of key glycolytic enzymes [77] (Figure 1). Notably, many of these proteins also play important roles in insulin signaling and converge on the PTEN/PI3K/Akt/mTORC pathway, which plays a central role in glucose metabolism, anabolic cell growth, proliferation, survival, metastasis, and drug resistance [70,78,79]. As such, therapies that target this pathway have been explored for the treatment of this malignancy (as reviewed [79]).

Recent studies have revealed that mutations in the exonuclease domain of the DNA polymerase POLE confer a favorable prognosis in EC [80]. Mutations in POLE have been linked to higher expression of glycolytic enzymes, including phosphoglucose isomerase (PGI), which converts glucose-6-phosphate to fructose-6-phosphate [81]. However, it is unclear how mutations in POLE regulate the expression of glucose metabolizing enzymes, and whether these alterations influence disease progression or response to treatment.

Epigenetic alterations may also be responsible for the development of EC [82], and hyperglycemia is known to impact epigenetics; a phenomenon known as hyperglycemic memory. In a murine breast cancer model, chronic hyperglycemia altered the epigenome of breast cancer cells, leading to activation of oncogenic pathways and increased tumor growth, even after the breast cancer cells were implanted back into euglycemic mice [83]. Whether hyperglycemic memory exists in EC, and contributes to EC pathology, remains to be determined.

### 2.2. Increased Expression of Glucose Transporters in Endometrial Cancer

Primary and recurrent endometrial tumors can be detected by positron emission tomography (PET) using a radiolabeled analogue of glucose, [^18^F]fluorodeoxyglucose (FDG) [84]. This indicates that glucose uptake is higher in EC relative to surrounding tissues; a phenotype likely attributed to an increase in the expression of glucose transporters, known as GLUTs (Figure 1). For example, GLUT1 expression is elevated in EC [85,86,87,88,89,90,91] and increases with tumor stage [85]. In a few cases (4%), increased expression of GLUT1 may be attributed to gene amplification [86]. It is thought that GLUT1-mediated glucose uptake may be driven by GLUT1 localization at the plasma membrane, which has been identified in EC but not in the inactive endometrium or endometrial hyperplasia [89]. In support of this, previous studies have shown that the maximum standardized uptake value (SUVmax) of ^18^F-FDG correlated with GLUT1 expression and histological grade in EC [92], highlighting an important role for GLUT1 in mediating glucose transport in EC. However, other GLUTs that have different affinities for glucose than GLUT1 (*Km* = 3 mM), may also contribute to increased glucose metabolism in EC. Our laboratory has shown that GLUT6 (proposed *Km* = 5 mM) is upregulated in low grade/stage endometrioid cancers and human EC cell lines (HEC-1A, Ishikawa, MFE-296, MFE-319, AN3CA, RL95-2, and KLE cells) compared to non-malignant endometrium and cell lines (MAD11 stromal cells and hUE-Ts epithelial cells), respectively [91]. Functional studies revealed that knockdown of GLUT6 expression in EC cell lines (MFE-296, RL95-2) inhibited glucose uptake (at physiological glucose concentrations of 5 mM), reduced glycolysis, and induced cell death [91]. Other glucose transporters that may contribute to increased glucose uptake in EC are GLUT8 (*Km* = 2 mM) and GLUT3 (*Km* = 1.5 mM). A study has shown a trend for increased GLUT8 expression in poorly differentiated EC and uterine papillary serous carcinoma compared to well differentiated EC [85]. Similarly, GLUT3 mRNA expression increased with tumor grade (poorly differentiated tumors) and GLUT3 protein was detected in 30% of ECs [86]. Overall, these studies suggest that increased expression of GLUTs with differing affinities for glucose, may allow EC cells to capitalize on glucose availability under diverse conditions.

### 2.3. Increased Glucose Metabolism in Endometrial Cancer Cells

Glucose is metabolized through diverse cellular pathways, and the fate of glucose-derived carbons is regulated by various signaling proteins and enzymes. Typically, cancer cells and proliferating cells ferment glucose to lactate via glycolysis in the cytoplasm, even in the presence of oxygen. This process is termed ‘aerobic glycolysis’ or the ‘Warburg effect’. Although this type of metabolism it is not considered energetically favorable (with respect to ATP production) it is thought to benefit cancer cells by allowing glycolysis to continue (by cycling NADH back to NAD^+^) and diverting glucose-derived carbons into biosynthetic pathways that are essential for cell proliferation (as reviewed [93]). Research in our laboratory has demonstrated that glycolytic enzymes such as hexokinase 2 (HK2) and pyruvate kinase isozyme M2 (PKM2), as well as lactate dehydrogenase A (LDHA), are elevated in EC (low grade/stage endometrioid histologies) compared to adjacent non-malignant endometrium [91] (Figure 1). HK2 is the first rate-limiting enzyme of the glycolytic pathway and helps trap glucose inside the cell via phosphorylation producing glucose-6-phosphate (Figure 1). In contrast, PKM2 is the last enzyme of the glycolytic pathway that dephosphorylates phosphoenolpyruvate to produce pyruvate (Figure 1). LDHA then converts pyruvate into lactate and in the process inter-converts NADH to NAD^+^. Lactate can then be exported outside the cell, or back inside the cell, by proton-coupled monocarboxylate transporters, including monocarboxylate transporter 1 (MCT1) (Figure 1). High expression of MCT1 is associated with lower recurrence-free survival, cancer-specific survival and overall survival in EC [94].

A biosynthetic pathway exploited by cancer cells is the pentose phosphate pathway (PPP) which utilizes glucose-6-phosphate (from glycolysis) to support nucleotide biosynthesis, as well as antioxidant defense through the generation of reduced nicotinamide adenine dinucleotide phosphate (NADPH). NADPH serves as an electron donor for the production of reduced glutathione (GSH). GSH is utilized by enzymes including glutathione peroxidase (GSH-Px) which detoxifies hydrogen peroxide and lipid hydroperoxides, thus protecting cells from oxidative damage. GSH-Px activity and expression is up-regulated in EC tissues compared to endometrial tissue from age-matched healthy controls, and is associated with well-differentiated and less invasive cancers [95]. GSH-Px activity also increased in rat uterine tissue following exposure to exogenous estrogen and decreased in response to exogenous progesterone [95]. Thus, higher levels of glucose metabolism coupled with increased exposure to unopposed estrogen may benefit EC cells by promoting antioxidant defense via GSH production (Figure 1).

Following glycolysis, glucose-derived pyruvate can be further metabolized to acetyl-coenzyme A (CoA) via mitochondrial metabolism. Acetyl-CoA, along with NADPH, are essential substrates for de novo lipogenesis (Figure 1). Lipids produced by this pathway are important components of cell membranes and are required for lipidation reactions and cell signaling (as reviewed [70]). Research from our laboratory has shown that ECs (low grade/stage endometrioid histologies) expressed higher levels of key lipogenic enzymes including ATP citrate lyase (ACLY), acetyl-CoA carboxylases (ACC), and fatty acid synthase (FASN) [91] (Figure 1). Furthermore, our examination of TCGA datasets revealed that 40% of Type I and 68% of Type II ECs harbored alterations in glycolytic-lipogenic genes, and that patients with Type I EC (including obese women and those with stage II–IV cancers) that had this genetic signature had poorer overall survival [91]. Our studies also showed that most EC cell lines (HEC-1A, Ishikawa, MFE-296, MFE-319, AN3CA, RL95-2, but not KLE cells) had higher rates of glycolysis and lipogenesis (derived from glucose), compared with normal endometrial stromal (MAD11) and epithelial (hUE-Ts) cells [91]. EC cell lines harboring these metabolic phenotypes were highly sensitive to the lipogenesis inhibitor, TOFA, in vitro and the dual glycolytic-lipogenic inhibitor, 3-bromopyruvate, in vitro and in vivo [91]. Notably, our findings have been supported by other studies demonstrating that glucose transporters and the enzymes that regulate glycolysis and lipid synthesis are upregulated in EC [85,86,88,96].

A small proportion of glucose (2–5%) that enters a cell is also metabolized through the hexosamine pathway which generates uridine diphosphate *N*-acetylglucosamine (UDP-GlcNAc) (Figure 1) (as reviewed [97]). UDP-GlcNAc can be used for the common post-translational modification, *O*-Linked β-N-acetylglucosamine (*O*-GlcNAc), which occurs on serine or threonine residues of nuclear, cytoplasmic and mitochondrial proteins. This modification can impact the activity of oncogenes, and lead to the activation of oncogenic signaling pathways and metabolic pathways, such as the PPP (as reviewed [97]). A study has shown that mRNA expression of *O*-GlcNAc transferase (*OGT*) (which attaches *O*-GlcNAc to proteins) and Meningioma-Expressed Antigen 5 (*MGEA5*) (which removes *O*-GlcNAc from proteins) both increased with higher histological grade and were associated with greater myometrial invasion in EC [98] (Figure 1). Therefore, a high turnover of protein *O*-GlcNAcylation reactions may also contribute to aggressive phenotypes in EC as a result of increased glucose availability and metabolism.

Overall, high levels of systemic glucose may promote tumor growth by providing EC cells with a carbon source that can be utilized for diverse biosynthetic pathways that are essential for cell proliferation. Moreover, the proteins and enzymes that regulate glucose metabolism may prove viable therapeutic targets for this malignancy.

### 2.4. High Glucose-Induced Alterations in Endometrial Cancer Cells

Increased glucose availability has also been linked to other well-known cancer cell characteristics and oncogenic signaling pathways in EC. For example, EC cell lines (ECC-1, Ishikawa) cultured in physiological levels (5 mM) and high levels (25 mM) of glucose exhibited greater clonogenicity and adhesion, and lower phosphorylation of 5’ adenosine monophosphate-activated protein kinase (AMPK), compared with those grown in low glucose (1 mM) media [99]. These results suggest that glucose conditions above physiological levels may suppress AMPK activity in EC cells, which is typically associated with an increase in anabolic metabolism (Figure 1). In another study, EC cells (UECC, Ishikawa, RL95-2) cultured in high glucose conditions (25 mM) compared to physiological levels (5 mM) were more invasive, expressed higher levels of epithelial-mesenchymal markers (TWIST, SNAIL, CTNNB1), estrogen receptor α (ERα), and vascular endothelial growth factor receptor (VEGFR), and secreted more VEGF [100] (Figure 1). The EC cell line, Ishikawa, was also shown to express higher levels of signal transducer and activator of transcription 3 (STAT3), its regulators Janus kinases 1 (JAK1) and 2 (JAK2), and lower expression of forkhead box class O1 (FOXO1), when cultured in high glucose conditions (25 mM) compared to physiological levels (5 mM) [101]. Furthermore, high glucose-induced expression of many of these signaling proteins was abrogated following treatment with supraphysiological doses of metformin (10 and 20 mM) [101] (Figure 1). Thus, chronic exposure to high levels of glucose may contribute to sustained activation of multiple oncogenic signaling pathways contributing to EC development and progression (Figure 1). Whether these factors play a role in regulating glucose metabolism in EC in vivo, are yet to be defined.

### 2.5. Non-Enzymatic Use of Glucose in Endometrial Cancer

Sugars that can act as reducing agents, such as glucose, can also bind non-enzymatically with amino groups on proteins to form precursors of advanced glycation end-products (AGEs). AGEs are reactive molecules that cause damage to proteins, lipids and nucleic acids. AGE formation occurs naturally as part of the aging process but is accelerated in hyperglycemic conditions where glucose is more abundant [102], with the degree of protein binding proportional to the degree and duration of hyperglycemia [103]. Hyperglycemia results in the chronic accumulation of AGEs which when bound to the Receptor for AGE (RAGE) increase inflammatory signaling (by promoting NF-κB activation) and oxidative stress [104]. Studies have shown that RAGE expression is elevated in EC, particularly less differentiated tumors [105,106] and higher levels are associated with poorer overall survival [106]. Interestingly, AGE levels and RAGE expression are elevated in endometrial tissue from obese women compared to lean women [107]. Whether AGEs are present at higher levels in the endometrium of hyperglycemic women, and whether AGE-RAGE interactions and signaling play a role in EC development or progression, remains to be determined.

### 2.6. Potential Role for Glucose in the Endometrial Cancer Microenvironment

The microenvironment of endometrial tumors contains diverse cell types including stromal cells and immune cells. Many of these cell types can take advantage of glucose availability to support the progression of EC. In particular, the metabolism and polarization of tumor-associated macrophages (TAMs) can play a key role in promoting angiogenesis, migration/invasion, and resistance to therapy [108,109]. Increases in TAMs within the necrotic core have been associated with higher clinical stage, myometrial invasion and histological differentiation [110], while high levels of TAMs at the invasive margin have been associated with greater myometrial invasion and vascular space invasion in EC [111]. Like normal macrophages, TAMs are considered to polarize into the M1-like (anti-tumoral, pro-inflammatory) or M2-like (pro-tumoral, anti-inflammatory) phenotypes. M1-like TAMs are more glycolytic while M2-like TAMs are more reliant on oxidative metabolism (OXPHOS). However, there are conflicting reports as to the metabolic phenotype of tumor-promoting TAMs in different malignancies (as reviewed [108]). Some have indicated that hypoxia, or lactate produced by glycolytic cancer cells, may stimulate M2 polarization of TAMs due to stabilization of HIF1α, while other studies suggest that tumor-promoting TAMs are themselves are more glycolytic (M1-like) and subsequently produce high amounts of lactate which can promote angiogenesis (as reviewed in [108,109]) (Figure 1). Regardless, TAMs and the metabolism of glucose by these cells or other cells in the microenvironment may play a key role in EC progression.

A recent study in mice has provided novel insight into how hyperglycemia may impair intestinal barrier permeability by causing altered gut microbiota composition (dysbiosis), leading to systemic dissemination of microbial products and inflammation [112]. Levels of HbA1c, but not BMI and other hallmarks of metabolic disease, showed the strongest correlation with serum levels of microbial pattern recognition receptor (PPR) ligands, suggesting that chronic hyperglycemia may contribute to intestinal barrier dysfunction and gut-related systemic inflammation in humans. Interestingly, a recent study has shown that uteri from EC patients have different microbiomes to those without cancer [113]. Specifically, microbiome sequencing of DNA (16S rDNA V3–V5 region) found significant differences in ɑ- and β-diversity in the uteri of patients with EC compared with those that had benign uterine conditions [113]. Twelve taxa were enriched in the EC samples (from Firmicutes, Spirochaetes, Actinobacteria, Bacteroidetes and Proteobacteria phyla) and the combined presence of *Atopobium vaginae*, an uncultured *Porphyromonas* species, and high vaginal pH (>4.5) were associated with EC [113]. Whether hyperglycemia also influences the uterine microbiome, or contributes to an inflammatory environment in EC, remains to be determined.

## 3. Targeting Hyperglycemia: Opportunities for Improving Survival and Reducing EC Risk

### 3.1. Anti-Diabetic Agents

Metformin is a biguanide commonly used to treat Type 2 Diabetes Mellitus (T2DM). In recent years, several studies have reported the potential benefits of metformin on EC incidence and outcomes [114,115,116]. A systematic review by Meireles et al. demonstrated that metformin was associated with reversal of atypical endometrial hyperplasia, decreased expression of cell proliferation biomarkers, and a higher overall survival rate among metformin-users compared to non-metformin users and non-diabetic patients [114]. This finding is corroborated by Perez-Lopez et al., who demonstrated lower overall mortality among postmenopausal women with EC following treatment with metformin [115]. Tang et al. also concluded that metformin use resulted in lower EC incidence and significantly improved the survival of EC patients [116]. However, it is still unclear whether treatment with metformin improves overall survival for women with EC. Some retrospective cohort studies suggest an improvement in overall and recurrence-free survival [117,118,119,120], while others found no such benefit [121,122,123,124]. Women undergoing fertility-sparing management of atypical hyperplasia and early EC may benefit from adjuvant metformin therapy [125,126,127,128,129] and a large randomized control trial in this area is currently underway [130].

It has been proposed that metformin can exert both direct and indirect antineoplastic effects. The direct effect of metformin revolves around the negative regulation of key cellular growth and proliferation signaling pathways, in particular the AMPK/TSC2/mTOR/S6 pathway [131,132] (Figure 1). Our research group and others have shown that supraphysiological concentrations of metformin (≥1 mM) are required to inhibit EC cell viability [91,133,134,135]. At these doses, metformin is shown to induce G0/G1 cell cycle arrest, apoptosis, autophagy, increase phosphorylation of AMPK and decreases phosphorylation of ribosomal S6 kinase; a downstream target of mTOR [133,134]. Another study showed that metformin (10 mM) potentially inhibits epithelial-mesenchymal transition (EMT) in EC cells (Ishikawa and KLE) by increasing E-cadherin expression and decreasing N-cadherin, Snail and Slug expression [136]. Additionally, metformin raised the expression of βKlotho, an essential co-receptor of fibroblast growth factor (FGF) receptor complexes, and inhibited the phosphorylation of the downstream target, ERK1/2, in Ishikawa cells [136]. Interestingly, some studies have reported that metformin may impede tumor progression by reducing TAMs or inducing M1-like polarization of TAMs [137,138,139], while others indicate an anti-inflammatory effect on stromal cells [140]. Whether metformin also exerts these effects in the tumor microenvironment of EC remains to be determined.

Therapeutic doses of metformin result in only micromolar concentrations in plasma and various tissues, including target organs such as the liver [141], suggesting that any beneficial effects of metformin on EC growth are indirect. In further support of this, a small prospective study involving 31 patients found that preoperative therapy of metformin administered at a therapeutic dose (1500–2250 mg/day for 4 weeks) was associated with reduced Ki67 and topoisomerase IIɑ markers, coupled with an increase in the phosphorylation of AMPK and p27, and a decrease in phosphorylation of S6 kinase and ERK1/2, in EC tissues [135]. However, metformin was only detected at low micromolar concentrations in EC tissues, leading the authors to suggested that the antiproliferative effect of metformin was due to indirect effects, i.e., lowering of blood glucose, insulin, IGF-1, and leptin levels [135]. A follow-on study showed that treatment of 5 EC patients with therapeutic doses of metformin led to a decrease in the expression of the long non-coding RNA, *H19*, in EC tissues which was attributed to increased methylation of the H19 gene, downstream of AMPK phosphorylation [142]. H19 plays an important role in promoting tumor cell migration and invasion. Recently, it was reported that nuclear-localized ERɑ decreased, while PR and Krüppel-like factor 9 (a transcriptional regulator of endometrial cell proliferation and differentiation) increased in glandular epithelial cells of obese non-diabetic women with EC treated with therapeutic doses of metformin (1000–1700 mg/day for 4 weeks), compared with obese non-diabetic women with EC who did not receive treatment [143]. However, markers of proliferation (Ki67) and apoptosis (TUNEL) were not different between the groups [143]. In contrast to the potential beneficial effects of metformin in EC tissues, one study has shown that therapeutic doses of metformin significantly reduced the expression of the tumor suppressor protein, protein phosphatase 2A (PP2A) and its regulatory subunit *PPP2R4*, in EC tissues compared with patient-matched tissues obtained prior to treatment [144]. Therefore, it is possible that these differing effects may, in part, contribute to the conflicting or inconclusive findings in retrospective cohort studies.

Sodium glucose cotransporter 2 (SGLT2) inhibitors are also used for the treatment of T2DM. These inhibitors work by selectively targeting renal SGLT2 to prevent glucose reuptake in the kidney, thus lowering blood glucose levels [145]. One study has shown that the SGLT2 inhibitor, dapagliflozin, slowed the growth of breast and colon cancer xenografts in an insulin-dependent manner, without lowering blood glucose levels [146]. Whether SGLT2 inhibitors will also inhibit tumor growth in preclinical models of EC, or in patients, is yet to be revealed.

### 3.2. Weight Loss and Bariatric Surgery

There is accumulating evidence that sustained weight loss can result in a reduced incidence of EC and mortality in obese women. Intentional weight-loss of >5% was found to be associated with a significantly reduced risk of developing EC in the Women’s Health Initiative (WHI) observational study [147]. The association was strongest in obese women who achieved weight-loss compared to non-obese women with stable weight (OR 0.44; 95% CI 0.25–0.78). A number of observational studies have subsequently compared bariatric surgery to non-surgical methods of weight loss and lifestyle change, showing that bariatric surgery is more effective in reducing the incidence of EC. Anveden et al. [148] analysed the long-term follow up of women in the prospective Swedish Obese Subjects (SOS) study [149] which matched 1420 obese women who underwent bariatric surgery with 1447 controls (age 37–65 years) who received ‘conventional’ obesity management at their primary health care center. At a median follow-up of 18.2 years, bariatric surgery was associated with a significantly reduced risk of EC (HR = 0.56, CI = 0.35–0.89, *p* = 0.014). Furthermore, a recent systematic review of bariatric surgery and subsequent risk of EC development [150] identified five prospective studies comparing a total of 113,032 women who underwent bariatric surgery to 848, 864 controls, finding a significant reduction in EC risk in the bariatric surgery group (OR = 0.317, 95% CI = 0.161–0.627).

However, it is unclear what the exact mechanisms are that effect the significant reduction in risk of EC following weight loss achieved by bariatric surgery. Any intentional weight loss of >10% body weight has been shown to favorably modulate serum levels of not only plasma glucose and insulin, but also inflammatory markers (CRP, IL-6, TNF α), hormones (SHBG, FSH and LH), and adiponectin [151,152]. In the Swedish Obese subjects study, the reduction in EC risk with bariatric surgery was significantly associated with plasma insulin and glucose levels [148], supporting hyperglycemia as a target (directly or indirectly) for treatment. In a study by Modesitt et al., 20 obese women who underwent bariatric surgery had significantly lower levels of glucose, increased levels of chiro-inositol, and alterations in metabolites indicating reduced inflammation, in post-surgery bloods compared with pre-surgery bloods [153]. In contrast, estrogen and progesterone levels were not significantly altered by surgery. These results suggest that bariatric surgery improves glucose homeostasis, insulin sensitivity, and reduces inflammation. Interestingly, in this study, of the women who retained their uterus at the time of bariatric surgery, 10% had endometrial hyperplasia which appeared to resolve with weight loss alone [153]. Therefore, it is possible that lowering of blood glucose levels may be a key factor in reducing the risk of EC in obese women undergoing bariatric surgery.

## 4. Conclusions

EC is the most common gynecological malignancy in developed countries and the incidence of this malignancy is increasing. Therefore, it is important that we understand the etiology and pathogenesis of this disease to reduce EC risk and incidence, and improve outcomes following treatment. This review has highlighted the relationship between hyperglycemia and EC risk (Table 1). As such, there is evidence to suggest that chronic elevation of blood glucose may increase the risk of EC, independent of obesity. Although it is to be noted that the association between hyperglycemia and increased risk of EC is not supported in all studies [21,26] (as summarized in Table 1). Nevertheless, precancerous and cancerous endometrial cells, as well as cells in the endometrial tumor microenvironment, may exploit this excess availability of glucose to fuel anabolic pathways and disease progression. The genetic landscape of EC indicates that most of these malignancies already have a pre-deposition for increased glucose metabolism through loss of PTEN and/or activation of the PI3K/Akt pathway via other mechanisms. This means that it is possible that humoral factors that regulate this pathway in normal cells, i.e., insulin, could be dispensable for EC initiation and progression. However, it is likely that hormones such as insulin, estrogen, and progesterone, as well as inflammatory molecules such as TNF-ɑ, all play their part in the process.

Lowering blood glucose levels, using drugs (metformin) or surgery, represents a potential avenue by which to reduce EC development in women considered ‘at risk’ of developing this disease. However, it is still unclear whether drugs such as metformin will be useful for improving outcomes for women who already have this disease or for women undergoing fertility-sparing management of atypical hyperplasia and early EC. Further studies including randomized controlled trials with consistency in metformin dose and duration are needed. In addition, future studies determining whether SGLT2 inhibitors also influence EC incidence will be of great interest.

## Figures and Tables

**Table 1:** Hyperglycemia and Endometrial Cancer.

Author	Design	Population	Measure	Results
NNHSS Cohort * [24]	Prospective Cohort	24,460 women 130 EC cases	Non-fasting blood glucose	Overweight women 2.45 times more likely to be diagnosed with EC with baseline non-fasting serum glucose ≥5.6 mmol/L (RR, 95%CI 1.11–5.42). No difference in risk found in women with normal BMI.
EPIC Cohort [25]	Nested case-control	284 EC cases 546 matched control subjects	Pre-diagnosis blood glucose	Post-menopausal women 2.6 times more likely to be diagnosed with EC with higher baseline blood glucose (RR, 95%CI 1.46–4.66, *p* < 0.001). No difference in risk found in pre- or peri-menopausal women.
WHIOS Cohort [26]	Prospective Cohort	250 EC cases 465 randomly-selected controls §	Fasting blood glucose	Fasting serum glucose levels were not associated with EC.
Me-Can Cohort * [27]	Prospective Cohort	290,000 women 917 EC cases	Non-fasting blood glucose	Higher baseline serum glucose associated with EC in the two highest BMI quintiles (RR = 1.17, 95%CI 1.09–1.25). No association seen in lowest BMI quintiles.
AMORIS Cohort [28]	Prospective Cohort	230,737 women	Blood glucose (fasting and non-fasting)	Women with impaired glucose metabolism (6.1–6.9 mmol/L) were at 2 times increased risk of EC diagnosis than women with normal glucose metabolism (<6.1 mmol/L). Women with diabetes mellitus (≥7 mmol/L or recorded diagnosis) were 1.75 times more like to be subsequently diagnosed with EC (HRs, 95%CI 1.11–3.60 and 0.82–3.75 respectively)
Alberta Population [29]	Case-Control	541 EC cases 961 age-matched controls	Fasting blood glucose	Small association between higher baseline blood glucose and EC diagnosis (OR = 1.15, 95%CI 1.00–1.31)
SEER Medicare database [30]	Case-Control	16,323 EC cases 100,751 controlsAll women ≥65 years old	Impaired fasting glucose as recorded in medical notes, including type 2 diabetes diagnosis	EC risk was associated with impaired fasting glucose (OR = 1.38, 95%CI 1.29–1.42)
Vasterbotten Intervention Project [31]	Prospective Cohort	33,293 women 117 EC cases with blood glucose measurements	Fasting blood glucose and blood glucose 2 h post 75g glucose load	Significant increasing trend in EC risk with increasing quartiles of fasting and post-load blood glucose with top versus bottom quartile RR of 1.86 (1.09–3.31, *p* = 0.019) and 1.82 (1.07–3.23, *p =* 0.028) respectively.
Modesitt et al. 2012 [32]	Case-control	38 morbidly obese women ≥50 years old scheduled for hysterectomy 22 with EC	Fasting blood glucose on morning of surgery	Significantly higher mean blood glucose in EC cases than controls (6.64 mmol/L cases vs. 5.04 mmol/L controls, *p* = 0.049)
Shou et al. 2010 [33]	Retrospective cohort	123 EC cases 90 age-matched controls	Fasting blood glucose	Significantly more cases than controls with blood glucose ≥ 5.6 mmol/L (50.4% vs. 27.8%, *p* < 0.05).
Zhan et al. 2013 [34]	Case-control	206 EC cases 350 controls	Pre-operative fasting blood glucose or type 2 diabetes diagnosis	Significantly higher mean blood glucose in EC cases than controls (6.2 vs. 5.4 mmol/L, *p* < 0.001).
Ozdemir et al. 2015 [35]	Case-control	199 women undergoing endometrial curettage for abnormal uterine bleeding 146 with normal endometrium 53 with hyperplasia or carcinoma	Fasting blood glucose	Significantly higher mean blood glucose in cases than controls (125.8 vs. 97.8 mg/dL, *p* < 0.001). Odds ratio of endometrial pathology according to fasting glucose level >88 mg/dL (4.9 mmol/L) was 0.11 (95%CI 0.03–0.3, *p* < 0.001).
Nead et al., 2015 [21]	Mendelian Randomization (MR) analysis	1287 case patients and 8273 control participants from EC studies in Australia and UK	Genetically-predicted fasting glucose levels using 36 genetic variants associated with fasting glucose	Genetically-predicted higher fasting glucose levels were not associated with EC (OR = 1.00, 95% CI = 0.67 to 1.50, *p* = 0.99).
Karaman et al., 2015 [36]	Case-control, retrospective	35 surgically staged EC patients 40 healthy controls	HbA1c levels within 3 months of hysterectomy	Significantly higher mean HbA1c in cases than controls (6.19% vs. 5.61%, *p* = 0.027).
Miao Jonasson et al., 2012 [37]	Prospective Cohort	25,476 patients with type 2 diabetes 183 cases of female genital cancer	Baseline HbA1c	No increased risk of female genital cancers with HbA1c ≥7.5% versus <7.5% No endometrial cancer-specific data.
Traviar et al., 2007 [38]	Prospective Cohort	25,814 women 13 EC cases Patients with a previous diagnosis of diabetes mellitus were excluded	Baseline HbA1c	4.05 –fold increase with baseline HbA1c 6.0–6.9% (HR, 95%CI 1.10–14.88) and 5.07 –fold increase with baseline HbA1c ≥7.0% in EC risk (HR, 95%CI 1.20–21.31) compared to HbA1c <6.0%
Levran et al., 1984 [39]	Case-control	22 EC cases 939 controls of similar weight	HbA1 1-10 years after diagnosis	HbA1 was significantly increased in cases compared to controls (*p* < 0.01)

* overlapping populations. § Diabetics and patients with blood glucose > 125 mg/dL (~6.9 mmol/L) were excluded from study; Blue shaded rows indicate studies showing a relationship between EC risk and increased blood glucose levels, whereas uncolored rows show no association between these factors.
